# Awareness of Breast Cancer Screening among the Medical and General Population of the North Region of Cameroon

**DOI:** 10.1155/2021/6663195

**Published:** 2021-07-27

**Authors:** Richard Tagne Simo, Erika Myriam Baiguerel, Armel Hervé Nwabo Kamdje, Paul Faustin Seke Etet, Mohamadou Ahmadou, Charlette Nangue, Phelix Bruno Telefo

**Affiliations:** ^1^Department of Biomedical Sciences, Faculty of Science, University of Ngaoundere, Cameroon; ^2^Department of Physiological Sciences and Biochemistry, FMBS, Garoua, University of Ngaoundere, Cameroon; ^3^Anatomo-Cytopathology Laboratory, University Hospital Center of Yaounde, Cameroon; ^4^Department of Biochemistry University of Dschang, Cameroon

## Abstract

Breast cancer has become a real public health problem in Cameroon, particularly in rural areas due to late diagnosis, resulting partly from the absence of national screening programs. This work is aimed at assessing breast cancer awareness in the North Region of Cameroon. Participants were selected in six health centers surrounding the rural area of Garoua, North Region, Cameroon, and administered a questionnaire aimed at assessing their awareness about breast cancer risk factors and screening. Out of the 475 women (including 37 medical personnel) interviewed, 45.5% attended at least secondary school; 91.3% were aware of the disease with the main sources of information from those around them (64.8%), media (46.5%), and health professionals in health facilities (42.7%). 23.3% had misconceptions and myth-based ideas on the origin of the disease. Ignorance was the main reason preventing the performance of breast self-examination, and the high cost prevents individuals from going for mammography. The highest awareness rate was observed in employed women with higher level of education. Our study highlights the need to raise awareness among the populations in North Region, Cameroon, about the risk factors and clinical signs of breast cancer and the importance of screening practice for early diagnosis of breast cancer.

## 1. Introduction

Breast cancer is a major public health challenge worldwide, as it is the leading cause of cancer-related deaths among women, particularly in developing countries [1, 2]. About 2.1 million women were diagnosed with breast cancer in 2018, and 626,679 breasts cancer-related deaths were reported. These represented an increase rate of 19% in the incidence and 17% in the mortality rates as compared to 2012 [1, 3–5]. In Africa, breast cancer represented 16% of cancer incidence and 18.3% of cancer-related mortality in 2018. In Cameroon, the situation is becoming alarming as breast cancer-related mortality rate displayed an increase of 34% between 2012 and 2018. 3,273 new cases were diagnosed and 1,780 women died of breast cancer in 2018, ranking it the cancer with the highest incidence and highest death toll in Cameroon [1, 6]. Also alarming is the fact that the number of women younger than 35 diagnosed with severe high-grade breast cancer has been increasing [7–9]. The five-year median survival rate is 22%, and most patients die within 12 months after diagnosis [10–13].

Breast cancer generally has a favorable prognosis in developed countries, partly due to detection at early and asymptomatic stages, thanks to the establishment of screening practices and therapeutic advances [14, 15]. Tools like the widely recognized Breast Cancer Risk Assessment Tool (BCRAT or Gail model) that were developed and adapted for the disparity in racial and ethnic groups in developed countries, in particular in the United States, have been proving to be challenging to adapt to developing countries [16, 17], including Cameroon, where information about the epidemiology and diversity of dominant risk factors of breast cancer in female populations is often scarce [8, 18].

In Cameroonian cities, few women attend national screening programs when available, partly due to the lack of awareness about breast cancer and screening methods [19, 20]. In addition, unfortunately, mammography is still inaccessible to the majority of the population due to high costs. Thus, cases with breast lumps are detected most often accidentally and more than 80% of cases are diagnosed in late stages (stages III and IV) where the disease usually has a poor prognosis [7, 9]. This explains why, in most African countries like Cameroon, breast cancer survival rate is low. Thus, breast cancer screening among population should be a priority in this country, particularly in rural areas where healthcare centers are lacking [21–23], considering that breast cancer incidence is expected to be different as compared to cities due to environmental differences [24–26].

The present study was aimed at assessing breast cancer awareness in the North Region of Cameroon, to contribute to the identification of the factors underlying the increased incidence and late diagnosis of breast cancer observed recently in Cameroon.

## 2. Materials and Methods

### 2.1. Participants and Ethical Considerations

Our study participants were 475 women randomly selected among the residents of the rural areas around the city of Garoua, North Region, Cameroon. Signed informed consent was obtained from each participant at recruitment. Before analysis, data collected were anonymized to protect the privacy of participants. All the procedures of the study were approved by the North Regional Delegation of the Ministry of Health.

### 2.2. Study Procedures

From August to December 2019, a cross-sectional and descriptive study was conducted in six health facilities of the North Region of Cameroon. Participants were recruited in the health facilities using simple random sampling. Women previously diagnosed with breast cancer and women undergoing unilateral or bilateral mastectomy were not included.

After being presented individually with the study rationale and detailed information on procedures, each participant willing to join the study had to sign an informed consent form. Afterwards, she was administered a questionnaire which was aimed at assessing awareness about breast cancer and screening practice and its risk factors and collecting some sociodemographic data. Then, physical breast examination was performed.

### 2.3. Data Collection

Awareness about breast cancer, associated risk factors, and recommended screening methods was assessed using a questionnaire collecting the following information:

- Demographic characteristics like age, education, and occupation
- Physiological factors with established risk potential in breast cancer context such as age (to assess whether age was advanced), age at first menarche (to determine early onset), and the presence and onset of menopause (late vs. early)
- Personal history and family-related factors such as parity (to distinguish nulliparous from multiparous), age at first pregnancy (to determine whether it occurred late), history of breastfeeding, history of exposure to ionizing radiation, history of hormonal treatment, history of OCP use, family history of breast cancer, and history of alcohol and tobacco consumption

In addition, the questionnaire also included questions aimed at assessing

- the awareness about the recommended screening methods for breast cancer early detection (breast self-examination and mammography)
- knowledge about commonly reported breast cancer symptoms like nipple discharge, change in skin appearance, change in breast size, the presence of breast masses, and inflamed axillary lymph nodes

### 2.4. Breast Physical Examination

When a nodule was identified as well as its characteristics (site, number, consistency, size, mobility relative to deep skin and plans, and painful or not) during physical breast examination performed in the gynecology service of the health facility, fine-needle aspiration (FNA) was performed with the consent of the participant, using recommended standard clinical procedures. Each sample collected was mounted on a slide, processed for May Grünwald-Giemsa staining and analyzed using bright-field microscopy (magnifications 4x to 40x) at the Laboratory of Anatomo-Cytopathology of the University Hospital Center of Yaoundé. In case of cancer, Fisher's simplification of Black's nuclear grading scheme was used to determine the grade.

Alternatively, when nipple discharge occurred during the examination, characteristics were determined and recorded as well (aspect, uni- or bilateral, and uni- or multiorifice). Moreover, palpation of the armpits was also performed to assess eventual axillary lymph node inflammation. The following characteristics of inflamed axillary lymph nodes were determined and recorded: number, size, and mobility relative to surrounding tissue.

### 2.5. Statistical Analysis

Data analysis was performed using XLStat Version 2019. The *χ*^2^ test was used to compare proportions, and correlations were determined between education, occupation, and breast cancer awareness and also between education, occupation, and breast cancer screening awareness and its practice. Statistical significance was set at *p* < 0.05.

## 3. Results

### 3.1. Sociodemographic Information

475 women were involved in this study, of which 37 (7.7%) were health professionals.  Table 1 shows the sociodemographic characteristics of participants. The age range was 13 to 72 years old, and the median was 42.5. Most participants were younger than 35 (84.6%), had attended at least secondary school (56.6%), and were not housewives (63.2%) (Table 1).

### 3.2. Awareness about Breast Cancer Risk Factors and Early Signs

Table 2 presents the distribution of participants according to their knowledge about breast cancer: 91.3% had heard about breast cancer and their main sources of information were their entourage (64.8%), media (46.5%), and health professionals (42.7%) (Table 2). More than 50% of the population was not aware of breast cancer risk factors. Most of them had misconceptions and myth-based ideas such as traditional breast massage (10.1%) and extended wearing of bra (13.2%) as the origin of the disease (Table 2). Hereditary factors (12.8%) and consumption of alcohol and tobacco (12.4%) were the most known actual risk factors of breast cancer (Table 2). Breast lumps (43.7%), pain (36.2%), and wounds (24.4%) were the most known breast cancer symptoms (Table 2).

### 3.3. Awareness about Breast Cancer Screening

Table 3 shows the distribution of participants according to their knowledge of breast cancer screening and the frequency of practice. 56.8% were aware of breast cancer screening and named clinical examination of the breast (34.9%), BSE (27.7%), and mammography (21.2%) as screening tools (Table 3). However, only 4.6% already performed mammography and 24.0% practiced BSE, of which 31.5% (7.5% of the overall participants) do so regularly (Table 3).

### 3.4. Factors Affecting Breast Cancer Awareness and Screening Practice Frequency

Participants had to choose among 10 choices on risk factors of breast cancer (including two erroneous concepts) and 5 choices on clinical signs/symptoms and the screening tools.  Table 4 shows the association between occupation and educational level of participants and their knowledge regarding breast cancer. Highly educated women were more likely to identify breast cancer actual risk factors (Table 4). However, they also had misconceptions and myth-based ideas: of the 48 women who attended university, 14 (29.1%) and 13 (27.0%), respectively, mentioned traditional breast massage and extended wearing of tight bra as risk factors and 15 (31.2%) had no idea (Table 4). Similarly, employed women were more aware of the disease and its risk factors and symptoms as compared to other groups (*p* < 0.0001) for most of the risk factors and symptoms (Table 4). Health professionals displayed better knowledge about breast cancer, although a few also had erroneous ideas and misconceptions (Table 4). Participants with higher education and employed women were also more aware of breast cancer screening, including breast self-examination (BSE), mammography, and ultrasound (*p* < 0.0001 vs. other groups) (Table 4).

In addition, the factors affecting breast self-examination (BSE) practice among the study participants are presented in Table 5. Breast cancer screening and BSE were mostly performed by women with higher education and employed women (*p* < 0.0001 vs. other groups) (Table 5).

### 3.5. Breast Examination Findings

Table 6 shows the results of the breast examination. Out of 475 participants, 329 agreed to undergo breast clinical examination as part of breast cancer screening. The following cases were detected: 23 with lumps, 3 with inflamed axillary lymph nodes, 2 with wounds on the breast, and 2 with wounds on the nipple (Table 6). Analyses of fine-needle aspirates of nodules and lesions revealed 5 cases with the following cytological findings: a benign breast tumor, a galactophoric cyst, a cystic abscess, an abscess markedly swollen, and low chronic inflammation.

### 3.6. Reasons for Not Practicing Screening

Participants mainly claimed (*i*) a lack of awareness about breast cancer screening methods and fear of discovering that they are sick, (*ii*) lack of time for screening, (*iii*) that should it be breast cancer it could only result from witchcraft and thus they would not need hospitals to deal with it, and (*iv*) that they do not have any complaint and no history of breast cancer in their family, so there is no need for any exam. Other astonishing reasons proposed to reject screening included the following: (*i*) “Breast cancer results from keeping coins in bra and I never do so”; (*ii*) “This lump in my breast has always been there and never had a problem or any complaint with it, so there is no need for a test”; (*iii*) “I do not want to know whether my lump is or is not cancer, because it could result in stress, and I will be rejected by my husband if my breast is removed”; (*iv*) “No one in my family ever had breast cancer and I never had any complaint, that is why I will not perform any screening”; and (*v*) “I do not know how to perform BSE, nobody ever taught me.”

## 4. Discussion

Despite decades of laboratory, epidemiological, and clinical research, breast cancer incidence continues to rise [27, 28]. This study aimed at assessing breast cancer awareness in the North Region reveals the existence of poor awareness about breast cancer's risk factors and its screening methods among women in the North Region of Cameroon. More than 50% of the population had no knowledge about breast cancer risk factors. Most of them had misconceptions about traditional breast massage (10.1%) and extended wearing of bra (13.2%) as risk factors. These results are surprising, given that most of the study participants have heard about breast cancer (91.6%), out of which some were young and had the right education level to access more information (46.5% attended at least secondary school). This could be explained by the fact that many of them heard about the disease from those around them such as friends and family members (64.8%). Indeed, in traditional African public beliefs, breast cancer is a mystical disease resulting from supernatural causes, including witchcraft, curses, and divine punishment [12, 23, 29].

The clinical signs most known by women were the presence of a lump in the breast (43.7%), breast pain (34.0%), and skin ulceration on the breast (23.5%), as reported in previous studies [7, 30]. Indeed, due to poverty, in the North Region of Cameroon, patients generally consult when breast ulceration is observed or breast lumps are painful, and these signs usually occur when the disease is already at late stages [10–13], contributing to late detection of the disease and poor prognosis [7, 9].

In the present study, highly educated and employed women identified breast cancer risk factors more accurately than uneducated and unemployed women (*p* < 0.0001). However, misconceptions on the origin of the disease were also found here. Of the 48 women with university degrees, 15 (31.2%) had no idea about disease risk factors, and 14 (29.1%) and 13 (27.0%), respectively, mentioned traditional breast massage and extended wearing of tight bra as risk factors. These women revealed that their erroneous information came from their entourage, and they did not see the importance of verifying it before participating in the study. This further suggests a lack of public awareness about breast cancer and underlines the need for education about early diagnosis and better prognosis of the disease. Media could help in disseminating the right information, as they are a source of information for 46.5% of the participants. Health facilities could help as well, as 28.7% of the participants reported that the health education sessions in local languages held during prenatal consultations and immunization days in Cameroonian healthcare centers are their major source of medical information. Studies in comparable contexts showed that presentations in local languages by qualified health professionals attract a large audience [29, 31]. As expected, in this study, most health professionals interviewed (and all those working with breast cancer patients) were aware of breast cancer risk factors and listed the clinical signs of the disease accurately. However, only 24.32% practiced BSE regularly, 10.81% had already undergone mammography screening as a medical prescription, and none had ever undergone mammography as routine screening. This observation corroborates reports in other African countries [31, 32] and suggests that good knowledge does not imply better screening practices. This was explained by negligence or the high costs of mammography.

Similarly, several other participants reported that it is possible to detect breast cancer early and cited clinical breast examination (32.87%), breast self-examination (22.60%), and mammography as screening tools. However, a low level of practice of mammography screening (4.11%) and BSE (6.16%) was also observed here, also due to high cost of mammography, together with a lack of mastery of the breast self-examination (BSE) technique and the fear of actually discovering a disease sign, considered a harbinger of mastectomy and death [12, 19, 29]. Considering the alarming rates of disease also reported elsewhere in the country, the Cameroonian Government should make mammography, the current gold standard for breast cancer screening, available for free to rural populations, for instance, through screening campaigns. BSE, a low-cost screening option which proved to be effective in similar contexts [1, 33], could be examined and recommended to women during these campaigns as it is the case in various successful breast cancer screening programs worldwide [14, 17, 22, 34].

Surprisingly, 79 (16.63%) participants refused clinical examination in this study, as they feared discovering breast cancer or to show their breasts to the medical professionals performing the examinations due to religious reasons (to preserve their intimacy). This also justified the refusal of several participants to be sampled for cytopathological examination of the nodules, as they argued that they needed the consent of their husbands. Breast palpation coupled with fine-needle aspiration, a cost-effective cytopathological analytic technique efficient in this context [35], revealed nodules in 4.55% of the participants, confirming the need for breast cancer screening campaigns in Cameroonian rural areas like the North Region.

## 5. Conclusion

Our study highlights the need to raise awareness among the public about the risk factors and clinical signs of breast cancer and the importance of screening practice by both mammography and regular practice of BSE and CBE in the diagnosis of breast cancer at early stage and management of the disease. The Cameroonian health authorities should organize mammography screening campaigns for an early detection of asymptomatic cases of breast cancer, considering the positive implications for treatment outcome.

## Figures and Tables

**Table 1:** Sociodemographic characteristics of participants.

Variables		*N*	%
Age (years)	<35	403	84.6
	≥35	72	15.1

Education	No formal	95	20.0
	Primary school	111	23.3
	Secondary school	221	46.5
	University	48	10.1

Occupation	Housewife	175	36.8
	Student	118	24.8
	Officer	24	5.0
	Health professional	37	7.7
	Other, informal sector	121	25.4

**Table 2:** Breast cancer awareness and known risk factors and symptoms reported.

Variables		*N*	%
Awareness	Heard about breast cancer	434	91.3

Information source	Media	221	46.5
	Friends	308	64.8
	Health professionals	203	42.7
	School	71	15.7

Risk factors	None	233	49.0
	Hereditary	61	12.8
	Prolonged use of pills	29	6.1
	Alcohol and tobacco	59	12.4
	Age	13	2.7
	Obesity	9	1.8
	Prolonged exposure to the sun	16	3.3
	Low breastfeeding	23	4.8
	Low deliveries	7	1.4
	Traditional breast massage	48	10.1
	Extended wearing of tight bra	63	13.2

Symptoms	None	170	35.7
	Lump in the breast	208	43.7
	Pain in the breast	172	36.2
	Wound on the breast	116	24.4
	Changes in breast shape and appearance	108	22.7
	Nipple discharge	61	12.8

**Table 3:** Breast cancer screening methods cited and personal screening frequency.

Variables		*N*	%
Awareness	Heard about screening	270	56.8

Screening methods	None	32	6.7
	Clinical examination	166	34.9
	BSE^a^	132	27.7
	Mammography	101	21.2
	Ultrasound	89	18.7

Screening practice	Mammography	22	4.6
	BSE	115	24.2

BSE practice frequency	Never	30	6.3
	Sometimes	79	16.6
	Often	36	7.5

^a^BSE: breast self-examination.

**Table 4:** Associations between occupation, education, and breast cancer awareness.

Variables	Occupation (*n*)					Total	*p*	Education (*n*)				Total	*p*
	O1	O2	O3	O4	O5			E1	E2	E3	E4		
*Risk factors* (df = 437, *χ*^2^ = 91)													
None	110	52	7	4	60	233	0.0001^∗∗∗^	61	61	96	15	233	0.0002^∗∗∗^
Hereditary	5	20	7	21	8	61	0.0001^∗∗∗^	1	4	40	16	61	0.0001^∗∗∗^
↑Use of pills	2	9	4	10	4	29	0.0001^∗∗∗^	0	2	18	9	29	0.0001^∗∗∗^
Alcohol/tobacco	7	28	1	16	7	59	0.0001^∗∗∗^	0	4	43	12	59	0.0001^∗∗∗^
Age	2	2	2	5	2	13	0.0002^∗∗∗^	0	2	8	3	13	0.116
Obesity	2	1	1	5	0	9	0.0001^∗∗∗^	0	2	4	3	9	0.080
↑Sun exposure	3	5	1	4	3	16	0.079	0	1	12	3	16	0.023^∗^
↓Breastfeeding	4	6	1	10	2	23	0.0001^∗∗∗^	0	1	17	5	23	0.001^∗∗^
↓Parity	2	2	0	2	1	7	0.306	0	1	5	1	7	0.433
Breast massage	4	23	4	13	4	48	0.0001^∗∗∗^	0	3	31	14	48	0.0001^∗∗∗^
Tight bra wearing	4	29	8	13	9	63	0.0001^∗∗∗^	0	4	46	13	63	0.0001^∗∗∗^
*Symptoms* (df = 437, *χ*^2^ = 91)													
None	87	19	1	1	62	170	0.0001^∗∗∗^	53	65	52	0	170	0.0001^∗∗∗^
Breast lump	40	77	22	34	35	208	0.0001^∗∗∗^	9	22	134	43	208	0.0001^∗∗∗^
Breast pain	52	60	14	23	23	172	0.0001^∗∗∗^	15	29	100	28	172	0.0001^∗∗∗^
Breast wound	42	22	7	13	32	116	0.289	20	28	53	15	116	0.602
Br. appearance	27	32	10	16	23	108	0.0002^∗∗∗^	7	16	68	17	108	0.0001^∗∗∗^
Nipple discharge	9	25	6	9	12	61	0.0001^∗∗∗^	3	7	42	9	61	0.0001^∗∗∗^
*Screening awareness*	77	81	22	37	33	270	df = 12; *χ*^2^ = 74 *p* < 0.0001^∗∗∗^	25	47	154	44	270	df = 9; *χ*^2^ = 91 *p* < 0.0001^∗∗∗^
*Screening methods* (df = 8; *χ*^2^ = 51.0) (df = 6; *χ*^2^ = 97.7)													
BSE^a^	24	38	17	33	20	132	0.0001^∗∗∗^	6	15	74	37	132	0.0001^∗∗∗^
CBE^b^	65	31	8	22	40	166	0.028^∗^	22	39	83	22	166	0.056^∗^
Mammography	16	33	11	25	16	101	0.0001^∗∗∗^	4	13	56	28	101	0.0001^∗∗∗^
Ultrasound	17	33	11	16	12	89	0.0001^∗∗∗^	4	11	54	20	89	0.0001^∗∗∗^

^a^Breast self-examination; ^b^clinical breast examination. O1 = housewife; O2 = student; O3 = officer; O4 = health professional; O5 = other informal sector; E1 = no formal education; E2 = primary school; E3 = secondary school; E4 = university; *χ*^2^: ^∗^*p* < 0.05; ^∗∗^*p* < 0.005; ^∗∗∗^*p* ≤ 0.0001.

**Table 5:** Factors affecting BSE practice.

Variables	BSE practice (*n*)			Total (*n*)	*p* value/df/*χ*^2^
	Never	Sometimes	Often		
*Educational level*					<0.0001^∗∗∗^/9/47.1
None	77	9	4	95	
Primary	84	8	6	111	
Secondary	147	44	17	221	
University	20	18	9	48	
*Occupation*					<0.0001^∗∗∗^/9/47.1
Housewife	130	18	9	157	
Health professional	7	21	9	37	
Officer	14	7	3	24	
Students	86	20	10	116	
Other informal sectors	91	13	10	114	

*χ* ^2^: ^∗∗∗^*p* < 0.0001; df = degree of freedom.

**Table 6:** Clinical examination findings.

Clinical aspect of the breast	*N*	%
Normal breasts	296	89.9
Nodules	23	5.2
Axillary lymph nodes	3	0.9
Umbilical nipple	2	0.6
Nipple discharge	1	0.3
Wound on the breast	2	0.6
Wound on the nipple	2	0.6

## Data Availability

All data generated or analyzed during this study are included in this published article.
